# Genome-wide identification and expression analysis of the *JAZ* gene family in turnip

**DOI:** 10.1038/s41598-021-99593-2

**Published:** 2021-10-29

**Authors:** Kai Jia, Cunyao Yan, Jing Zhang, Yunxia Cheng, Wenwen Li, Huizhuan Yan, Jie Gao

**Affiliations:** College of Horticulture, Xinjiang Agricultural University, Ürümqi, 830052 Xinjiang China

**Keywords:** Plant signalling, Plant stress responses

## Abstract

*JAZ* is a plant-specific protein family involved in the regulation of plant development, abiotic stresses, and responses to phytohormone treatments. In this study, we carried out a bioinformatics analysis of *JAZ* genes in turnip by determining the phylogenetic relationship, chromosomal location, gene structure and expression profiles analysis under stresses. The 36 *JAZ* genes were identified and classified into four subfamilies (*ZML*, *JAZ*, *PPD* and *TIFY*). The *JAZ* genes were located on 10 chromosomes. Two gene pairs were involved in tandem duplication events. We identified 44 collinear *JAZ* gene pairs in the turnip genome. Analysis of the Ka/Ks ratios indicated that the paralogs of the *BrrJAZ* family principally underwent purifying selection. Expression analysis suggested *JAZ* genes may be involved in the formation of turnip tuberous root, and they also participated in the response to ABA, SA, MeJA, salt stress and low-temperature stress. The results of this study provided valuable information for further exploration of the *JAZ* gene family in turnip.

## Introduction

Jasmonic acid and its biologically active derivatives are referred to as jasmonates (JAs), which participate in plant defenses to insects and contribute to developmental and growth controls^1–3^. JAs were first identified when plants suffered from biotic stresses (e.g., mechanical damage, pests, and diseases)^4^. Afterward, numerous studies revealed their significant role in plant responses to abiotic stresses such as low temperature, high temperature, drought, heavy metal, and salt stresses^5^. Under stress treatment, JAs induce the gene expression of signal transduction pathways, thereby regulating plant responses to adversity^6^. The interaction of JAs with other phytohormones and thus the regulation of plant stress resistance has also become a hot topic of research^7^.

JA signaling pathway, including the biosynthesis and metabolism of signal transduction molecules, JA signaling, and downstream gene response, is a complex process involving many genes and proteins^5,8^. When plants are stimulated by the external environment, they synthesize large amounts of jasmonic acid, which is formed into the highly biologically active JA-Ile by the action of the adenylate-forming enzyme JAR1. JA-Ile binds specifically to the jasmonic acid receptor F-box protein COI1 (coronatine insensitve1)^9^. The JAS domain is involved in the binding of COI1 and MYC2. The ZIM domain containing the TIFY motif is involved in binding NINJA (novel interactors of JAZ)^10^. JAZ is also considered to be a component of the JA co-receptor^11^ and acts as a "repressor" in the JA pathway^12^. In the absence of JA-Ile, JAZ proteins interact with NINJA proteins^13^ to recruit the co-repressor TPL (topless), which allows JAZ proteins to interact with downstream transcription factors, such as MYC2, to inhibit the transcriptional activation of JA-responsive genes by MYC2. In the presence of JA-Ile, the JA-Ile accumulated in response to stress binds to COI1 and promotes direct binding of the COI1-JAZs complex, forming a complex and causing ubiquitination of JAZ proteins by the E3 ubiquitin ligase SCF^COI1^ (Skp/Cullin/F-box) complex, which eventually degrades the JAZ repressor through the 26S proteasome. The SCF^COI1^ complex is formed by the binding of COI1 to ASK1/ASK2, Cullin1, and Rbx1, which are important components mediating the JA signaling response. Among them, MYC2 not only participates in the activation of jasmonic acid signaling, but also regulates the termination of jasmonic acid signaling, and can interoperate with MTB (MYC2-targeted bHLH) to regulate jasmonic acid signaling^14,15^.

*JAZ* gene family has many members that are involved in the regulation of plant development, abiotic stresses, and responses to phytohormone treatments, each with a different biological function^16^. For example, Overexpression of the *OsJAZ9* gene improves rice (*Oryza sativa*) tolerance to potassium deficiency by changing JA level and JA signal transduction pathway^17^. Overexpression of the *GsJAZ2* gene in soybean (*Glycine max*) significantly enhanced the resistance of transgenic lines to saline stress^18^. Overexpression of *AtJAZ1* in *Arabidopsis* can enhance host resistance to *Spodoptera exigua*^19^. Overexpression of *OsJAZs* in rice can lead to malformations in floral organ development^20,21^.

Turnip (*Brassica rapa* L. subsp. *rapa*) is a crucial root vegetable belonging to the Brassica subspecies of the family Cruciferae. Turnips are very sensitive to environmental stress which seriously affect the quality and yield of the tuberous roots^22–25^. Despite extensive studies of the *JAZ* family in various plant species, including cotton, rice, tomato, soybean, and cabbage, *JAZ* family genes have not yet been identified in turnip^18,26–30^.

In this study, we used genomic resources to systematically identify members of the turnip *JAZ* gene family and investigated phylogeny, chromosome locations, evolutionary history, structural characteristics. Furthermore, we also analyzed expression patterns of *JAZ* genes after different abiotic stresses and phytohormone treatments. This study will be useful for functional studies of JAZs in turnip.

## Materials and methods

### Identification of the *B. rapa JAZ* family genes

The genome sequences and annotation files of *Arabidopsis thaliana*, *B. oleracea* var. *Capitata*, and *Brassica rapa* subsp. *rapa* were obtained from the TAIR database (http://www.arabidopsis.org/), CNGB database (http://db.cngb.org/search/project/CNP0000469/), and Turnip Genome Database in JBrowse website (https://www.bioinformatics.nl/brassica/index.html?data=bras_tp%2Fdata&loc=A01%3A11421217..17131178&tracks=DNA&highlight ), respectively^21^.

To find the *JAZ* family genes in turnip genome, we downloaded the Markov model (HMM) files corresponding to the TIFY domain (PF06200) and JAS domain (PF09425) from Pfam protein family database (http://pfam.sanger.ac.uk/)^31^. The former two HMM profiles were used to search the turnip protein database for target hits with the TIFY and JAS domain using HMMER 3.0 software. The candidate JAZ proteins with E-values < 1.0E−05 were selected.

The JAZ protein sequences of 18 *A. thaliana* and 48 *B. oleracea* obtained from previous studies were used as query sequences to blast against turnip protein sequences^30,32^. All non-redundant sequences with E < 1.0e−5 were selected as candidate JAZ proteins.

The candidate JAZ protein sequences obtained by the above two methods were combined and uploaded to NCBI CD-Search (http://www.ncbi.nlm.nih.gov/Structure/cdd/wrpsb.cgi) to confirm the conserved domain. The molecular weight (MW) and isoelectric point (pI) of each JAZ protein were analyzed with the online tool ExPASy (http://www.expasy.org). The subcellular locations were predicted using Plant-mPLoc (http://www.csbio.sjtu.edu.cn/bioinf/Cell-PLoc-2/).

### Analysis of conserved motif and gene structure

The BrrJAZ proteins were used to create multiple protein sequence alignments using MEGA 7 software with the default parameter setting MUSCLE method^33^. The Gene Structure Display Server (GSDS: http://gsds.cbi.pku.edu.cn) was employed to determine the exon/intron organization of turnip *JAZ* genes by comparing predicted coding sequences with their corresponding full-length sequences. The conserved motifs in the identified turnip JAZ proteins were identified by MEME (http://meme-suite.org/).

### Sequence alignment and phylogenetic analysis

To infer the evolutionary relationship among *A. thaliana*, *B. oleracea* var. *Capitata*, and *B*. *rapa*, the phylogenetic analysis was performed. Multiple JAZ protein sequences were aligned using MEGA 7 software with the default parameter setting MUSCLE method. Based on this result, the neighbor-joining phylogenetic tree was constructed, with 1000 bootstrap values.

### Gene location and collinearity analysis and gene replication analysis

The position information of *BrrJAZ* genes was acquired from the genomic sequence annotation. TBtools software was used for the mapping of *JAZ* genes in the corresponding chromosome^34^. MCscanX software was used to analyze the gene duplication events^35^. Ks (synonymous) and Ka (non-synonymous) substitution of each duplicated *JAZ* gene pairs were calculated using KaKs_Calculator 2.0. To exhibit the synteny relationship of the orthologous *JAZ* genes obtained from turnip and other selected species, the syntenic analysis maps were constructed using the Dual Systeny Plotter software.

### Expression analysis from RNA-Seq data

The Illumina RNA-seq data were downloaded from the NCBI (Accession number: PRJNA273340) to study the expression patterns of *BrrJAZ* genes that participate in the tuberous root development. The turnip cultivar “Chang Huang Man Jing” was used as plant material. Samples consisting of tuberous root tissues were collected on day 18 (the early stage before cortex splitting, ES), day 28 (the stage of cortex splitting, CSS) and day 64 (the stage of root thickening, RTS) after sowing. Additionally, every stage had two independent biological replicates. The gene expression level was calculated using the Fragments Per Kilobase per Million reads (FPKM) method.

### RT-qPCR analysis of *JAZ* genes of turnip under abiotic stress and exogenous phytohormone treatment

#### Plant growth and treatments

The seeds of turnip cultivar “Qiamagu” were purchased from Tian Di He Co., Ltd. (Urumqi, China). All the experimental research on plants were conducted according to the proper guidelines and legislation of national and international regulations. Seeds were sterilized using sodium hypochlorite (5%) for 15 min and then rewashed with distilled water for 15 min. Thereafter, seeds were placed on filter paper in 9-cm petri dishes filled with 5 mL distilled water to germinate. Germinated turnip seeds (1-mm radicle emerged from the seed coat) were planted in plastic pots (20 × 12 cm) with coconut fiber as the substrate. Every pot was planted with 3 seedlings. All pots were placed in the greenhouse where the temperature was maintained at 25℃ and the photoperiod was 16 h/8 h (day/night). Each pot was irrigated with 50 mL of 1/2 Hoagland nutrient solution every 3 days. Two-week-old (two leaves) turnip seedlings with uniform sizes were selected for different abiotic stresses and exogenous phytohormone treatments.

*Phytohormone treatment* The turnip seedlings were sprayed with 100 μmol/L salicylic acid, abscisic acid, and methyl jasmonate, respectively.

*Abiotic stress treatment* The turnip seedlings were irrigated with 100 mmol/L NaCl solution as salt stress treatment. The turnip seedlings were placed in the 4 °C incubators as low-temperature treatment.

There were three repetitions in every treatment, and each repetition consisted of 9 plants. After 24 h, the leaf samples of every treatment were taken and frozen in liquid nitrogen and stored at – 80 °C for RNA extraction.

#### Extraction of total RNA and analysis of gene expression

Total RNA of turnip leaves was extracted using Trizol Kit (Beyotime, China). The quantity and purity of RNA were estimated by nanodrop microspectrophotometer (Thermo Fisher Scientific Inc., Wilmington, DE, USA). First-strand cDNA synthesis was carried out by reverse transcription Kit (Takara, Japan) with gDNA eraser. The specific primers of *BrrJAZ* genes were designed using NCBI primer-blast tools.

The sequences, amplification length, and locations of each primer have been listed in Table S1, and the specificity of the amplification products was tested by agarose gel electrophoresis. Each reaction contained 1.0 μL of cDNA, 0.4 μL of forward and reverse primer (10 μM), 10.0 μL of 2× SYBR qPCR Master Mix (Biosharp, China), and 8.2 μL double-distilled H_2_O in a total reaction volume of 20 μL and was conducted in ABI 7500 Real-Time PCR System (Applied Biosystems, Foster City, CA, USA) with 3 technical replicates by using hard-shell PCR plates. The reaction conditions were as follows: 95 °C for 3 min, followed by 45 cycles of 95 °C for 10 s, 60 °C for 30 s, and 72 °C for 20 s. The 2^−ΔΔCT^ algorithm was used to analyze the relative gene expression levels. β-Actin of turnip was used as the internal control to normalize the expression of the target genes. Between phytohormone treated and control samples, statistical analysis to find significant differential expression was determined using a two-tailed Student’s t-test in SPSS version 19.0 (IBM, Chicago, IL, USA, https://www.ibm.com/analytics/spss-statistics-software).

### Research involving plants

Experimental research and field studies on plants in this work comply with the IUCN Policy Statement on Research Involving Species at Risk of Extinction and the Convention on the Trade in Endangered Species of Wild Fauna and Flora.

## Results

### Identification and chromosome mapping of *JAZ* genes in turnip genome

Based on the genome data of turnip, HMM search was carried out using the HMM profiles of the TIFY domain (PF06200) and JAS domain (PF09425) as queries against the local protein database. By retrieving the database, we detected 35 non-redundant sequences. Then, 36 and 37 homologous proteins were obtained according to the BLASTP search using 18 *A. thaliana* JAZ proteins and 36 *B. oleracea* JAZ proteins, respectively. Subsequently, all the candidate JAZ proteins were merged and scanned using NCBI-CDD for the identification of their conserved domains. Finally, a total of 36 non-redundant *JAZ* genes were identified in turnip, including 26 *JAZ*, 2 *PPD*, 5 *ZML*, and 3 *TIFY* genes (Table S2).

Basic information of nucleotide and amino acid sequences of the *BrrJAZ* genes was summarized (Table 1). Based on the chromosomal location and the subfamily classification, the 36 *JAZ* genes in *B. rapa* were renamed. The length of these JAZ proteins ranged from 112 (*Brr*TIFY2) to 364 (*Brr*TIFY3) amino acid (aa) residues with an average length of 248.75 aa. The molecular weight ranged from 12.02 to 39.68 kDa, and the pI values varied from 4.56 to 10.02. Subcellular localization prediction showed that all JAZ proteins were in the nucleus.

**Table 1:** Gene information of *JAZ* family in turnip.

Gene name	CDS length (bp)	Protein length (aa)	Isoelectric point	Molecular weight (kD)	Location
*BrrJAZ1*	1062	353	9.3	37.57904	Nucleus
*BrrJAZ2*	654	217	10.02	24.77349	Nucleus
*BrrJAZ3*	666	221	5.18	23.12203	Nucleus
*BrrJAZ4*	825	274	9.79	29.32915	Nucleus
*BrrJAZ5*	822	273	9.11	30.15781	Nucleus
*BrrJAZ6*	723	240	9.11	26.13947	Nucleus
*BrrJAZ7*	552	183	9.95	20.54572	Nucleus
*BrrJAZ8*	342	319	8.57	12.99775	Nucleus
*BrrJAZ9*	360	119	9.76	13.70472	Nucleus
*BrrJAZ10*	351	116	8.88	13.05794	Nucleus
*BrrJAZ11*	1008	335	9.51	35.86815	Nucleus
*BrrJAZ12*	828	275	9.34	31.05405	Nucleus
*BrrJAZ13*	765	254	9.85	27.30006	Nucleus
*BrrJAZ14*	675	224	9.18	24.51966	Nucleus
*BrrJAZ15*	639	212	9.11	23.49644	Nucleus
*BrrJAZ16*	729	242	10.01	25.88651	Nucleus
*BrrJAZ17*	915	304	9.5	34.13637	Nucleus
*BrrJAZ18*	738	245	9.2	26.81027	Nucleus
*BrrJAZ19*	393	130	9.85	14.97178	Nucleus
*BrrJAZ20*	780	259	9.49	28.31018	Nucleus
*BrrJAZ21*	804	267	8.85	29.66028	Nucleus
*BrrJAZ22*	402	133	9.62	15.41432	Nucleus
*BrrJAZ23*	957	318	9.83	35.50621	Nucleus
*BrrJAZ24*	900	299	9.06	33.19413	Nucleus
*BrrJAZ25*	612	203	6.91	21.18776	Nucleus
*BrrJAZ26*	555	184	9.95	20.61794	Nucleus
*BrrPPD1*	960	113	8.78	35.11241	Nucleus
*BrrPPD2*	978	325	9.12	35.79108	Nucleus
*BrrTIFY1*	1035	344	9.69	37.40851	Nucleus
*BrrTIFY2*	339	112	4.56	12.02021	Nucleus
*BrrTIFY3*	1095	364	8.79	39.68089	Nucleus
*BrrZML1*	933	310	6.05	33.58929	Nucleus
*BrrZML2*	843	280	6.18	30.68593	Nucleus
*BrrZML3*	927	308	6.86	33.44333	Nucleus
*BrrZML4*	906	301	6.31	33.25202	Nucleus
*BrrZML5*	900	299	5.79	32.46877	Nucleus

**Figure 1:**
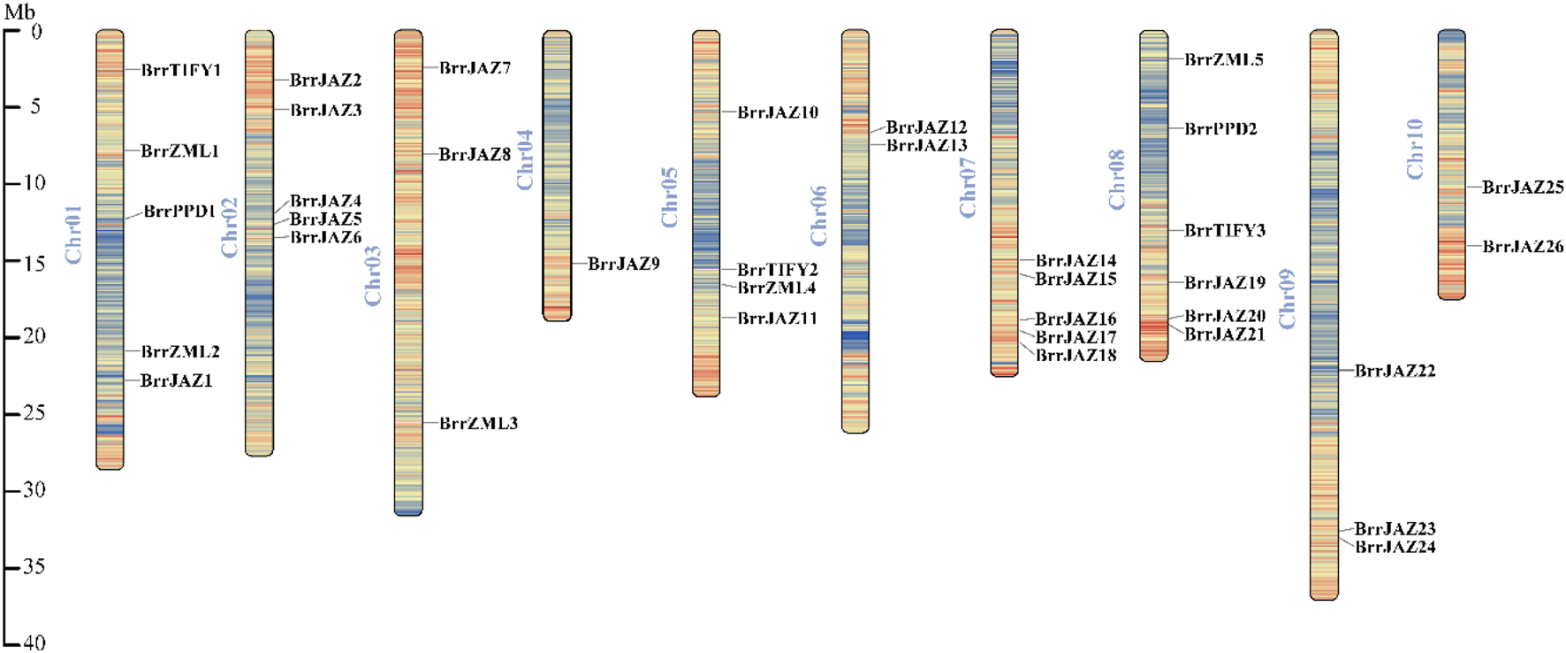
Distribution of *BrrJAZ* family genes in turnip. A total of 36 *BrrJAZ* genes were mapped to the 10 chromosomes according to their positions in the turnip genome. The chromosome number was shown on the left of each chromosome.

All 36 *JAZ* genes were assigned to ten chromosomes of *B. rapa* (Fig. 1), and the distribution of the *JAZ* genes on each chromosome was uneven. Chromosome 8 contained the largest number of *JAZ* genes (6 genes), followed by chromosomes 1, 2, and 7, which contained 5 genes. Only one *JAZ* gene was located on chromosome 4.

### Phylogenetic analysis of JAZ protein in turnip

**Figure 2:**
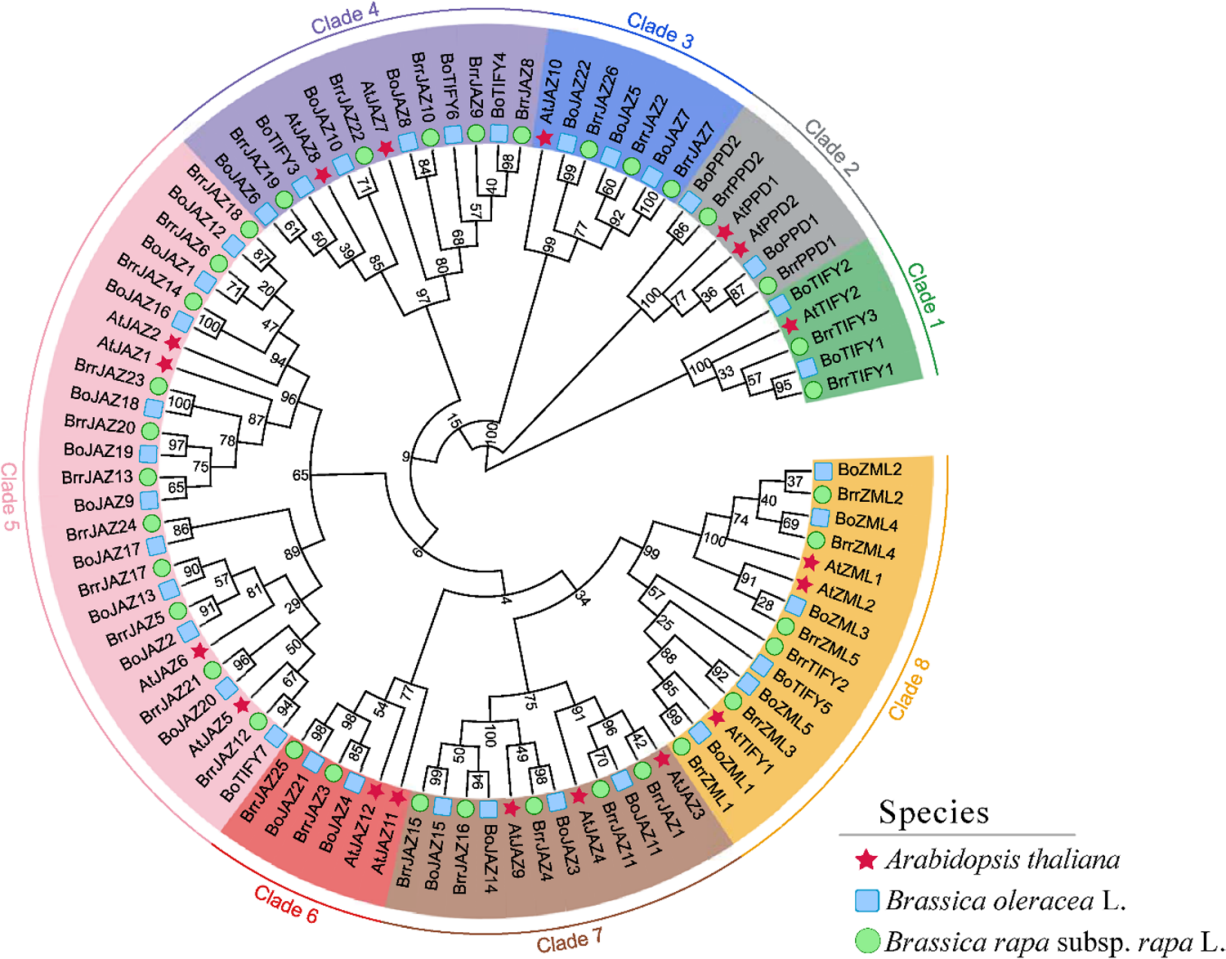
Phylogenetic relationship of JAZ protein sequences of turnip and its related species. The predicted full-length amino acid sequences of and 18 AtJAZ, 36 BrrJAZ, and 36 BoJAZ were used to construct a phylogenetic tree using MEGA 7 by the neighbor-joining method.

Based on the amino acid sequences of full-length JAZ proteins in *A. thaliana* (18), *B. oleracea* (36), and *B. rapa* (36), the phylogenetic tree was constructed using the neighbor-joining method in MEGA 7 software. The 90 JAZ proteins were grouped into eight clades (Fig. 2). Among these clades, Clade 1 was formed with 5 TIFY proteins (1 of *A. thaliana*, 2 of *B. rapa*, 2 of *B. oleracea*). Six PPD proteins (2 of *A. thaliana*, 2 of *B. rapa*, 2 of *B. oleracea*) were gathered together in Clade 2. Clades 3, 6, and 7 were three JAZ subfamily clades, including 7 (1 of *A. thaliana*, 3 of *B. rapa*, 3 of *B. oleracea*), 6 (2 of *A. thaliana*, 2 of *B. rapa*, 2 of *B. oleracea*), 12 (3 of *A. thaliana*, 5 of *B. rapa*, 4 of *B. oleracea*) members, respectively. Clade 4, 5, and 8 were mixed branches. Clade 4 was formed with TIFY (3 of *B. oleracea*) and JAZ proteins (2 of *A. thaliana*, 3 of *B. oleracea*, and 5 of *B. rapa*). Clade 5 was composed of 26 proteins, all of which were members of the JAZ except for the BoTIFY7 protein. 12 ZML (2 of *A. thaliana*, 5 of *B. oleracea*, and 5 of *B. rapa*) and 3 TIFY (each species possessed one TIFY protein) proteins were clustered in Clade 8.

### Gene structure and conserved motif analysis of *JAZ* genes in turnip

**Figure 3:**
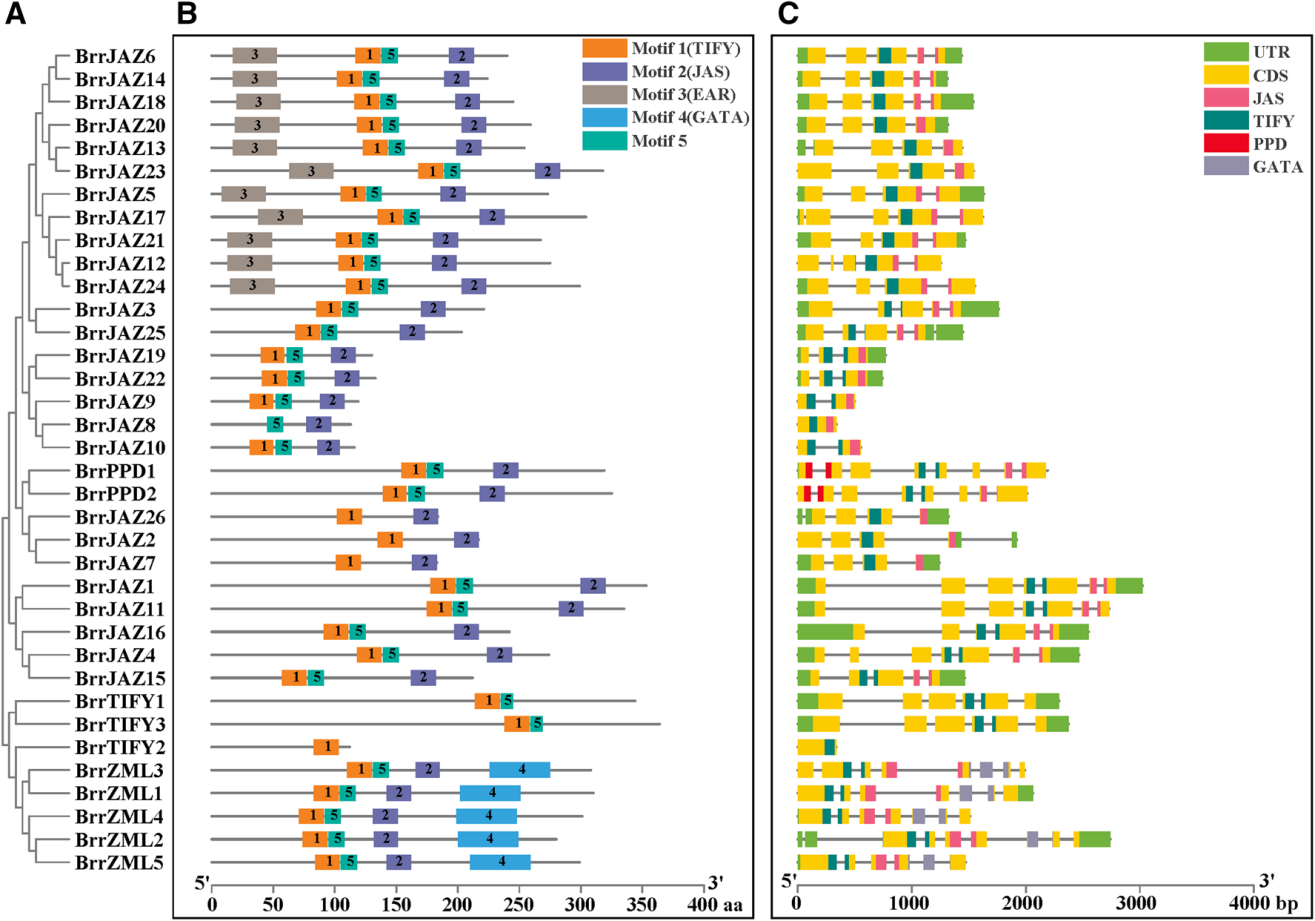
Gene structure, conserved motif and genetic relationship of JAZ protein in turnip. (**A**) Phylogenetic analysis of BrrJAZ proteins. The phylogenetic tree was performed in MEGA 7 with the neighbor-joining method. (**B**) The distribution of conserved motifs in BrrJAZ proteins. Each motif was represented by a colored box. (**C**) Exon/intron structure and conserved domains of *BrrJAZ* genes. Exons and introns were represented by yellow boxes and black lines, respectively. Each conserved domain was represented by a colored box.

**Figure 4:**
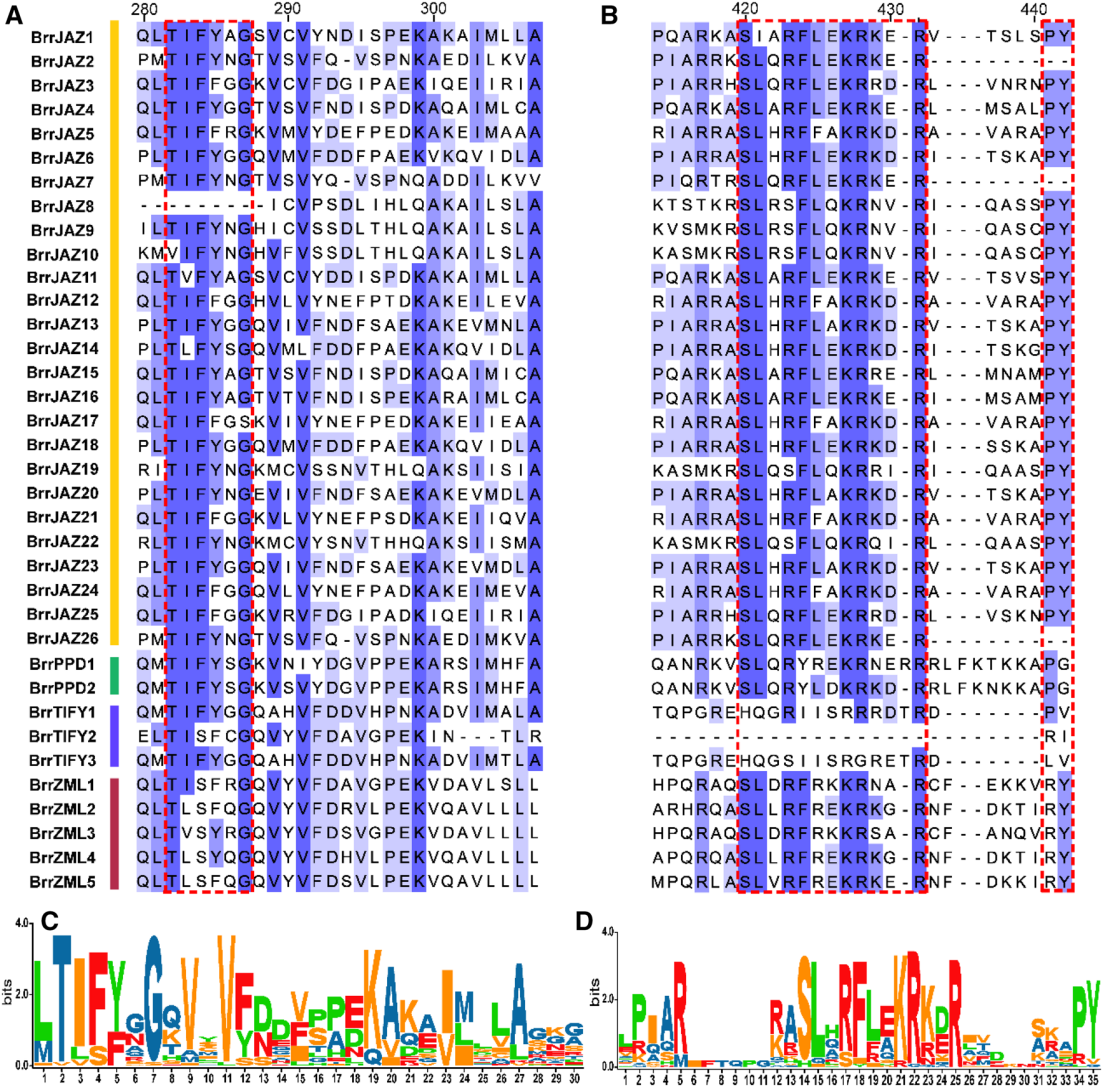
TIFY and JAZ domains in BrrJAZ proteins. (**A**) TIFY domain information. (**B**) JAZ domain information. (**C**) TIFY domain logos. (**D**) JAZ domain logos.

The phylogenetic relationships of the 36 *JAZ* family genes in turnip were closely related to their gene structures and motif compositions (Figs. 3, 4). The TIFY subfamily proteins did not contain the JAS domain. Five genes of the ZML subfamily clustered in one group, which contained similar motif compositions and gene structures, and their protein sequences contained the GATA structural domain. The 26 members of the JAZ subfamily all possessed the TIFY and JAS domains. The TIFY domain corresponded to motif 1. Motif 2 constituted the JAS domain. The EAR domain corresponded to motif 3. The GATA domain of ZML subfamily consists of motif 4 (Fig. 3B).

We compared the CDS sequences of turnip *JAZ* family genes and analyzed their exon–intron structures (Fig. 3C). The results showed that, among the *JAZ* family genes, *BrrTIFY2* and *BrrJAZ8* had the simplest gene structure, containing only one exon, whereas *BrrZML2* contained the highest number (9) of exons. *BrrZML2* had the highest number of introns (8).

### Gene duplication and collinearity analysis of JAZ family genes in turnip

**Figure 5:**
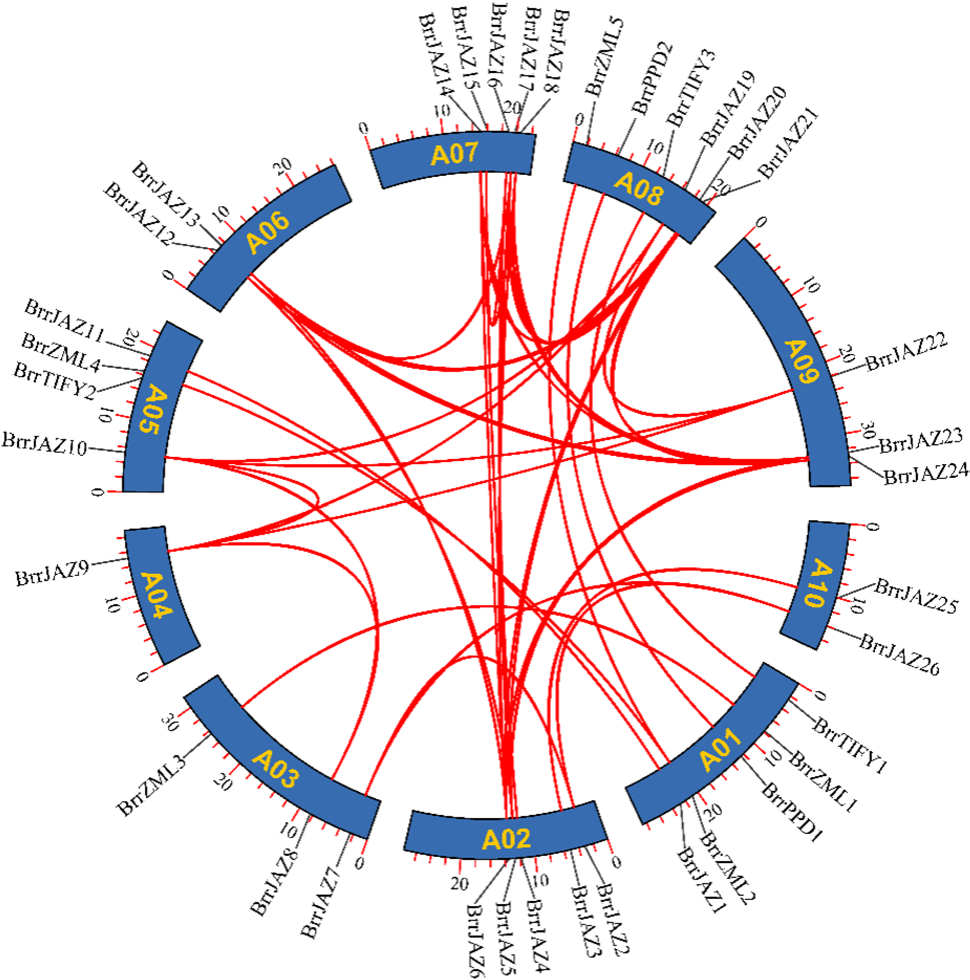
Collinearity analysis of *BrrJAZ* family genes. Red lines indicate duplicated *JAZ* gene pairs in the turnip genome.

Gene duplication events can lead to the expansion of gene families and play a crucial role in the adaptation by acquiring new gene functions. Given the importance of gene duplication in the evolution of plant gene families, we analyzed the duplication patterns of 36 *JAZ* family genes in the turnip genome, and 44 homologous duplicated gene pairs were identified (Fig. 5). Among these homologous duplicated gene pairs, *BrrJAZ14/BrrJAZ18* and *BrrJAZ15/BrrJAZ16* are two tandem duplicated gene pairs, while the other homologous gene pairs are formed by segmental duplication or whole-genome duplication. To estimate the evolutionary rates and selective pressure of the *JAZ* gene family in turnip, Ka and Ks analysis was subsequently performed (Table 2).

**Table 2:** Estimated Ka/Ks ratios of the duplicated *JAZ* genes in turnip.

Gene name		Ka	Ks	Ka/Ks
*BrrJAZ1*	*BrrJAZ11*	0.07986261	0.217796834	0.366683982
*BrrJAZ2*	*BrrJAZ7*	0.114915011	0.266962533	0.430453703
*BrrJAZ2*	*BrrJAZ26*	0.081516779	0.43741924	0.186358468
*BrrJAZ3*	*BrrJAZ25*	0.111963933	0.379043121	0.295385741
*BrrJAZ4*	*BrrJAZ16*	0.10009422	0.242448917	0.412846636
*BrrJAZ4*	*BrrJAZ15*	0.091004188	0.249788611	0.364324808
*BrrJAZ5*	*BrrJAZ12*	0.346141361	1.301435444	0.265968905
*BrrJAZ5*	*BrrJAZ17*	0.124648927	0.310670236	0.401225843
*BrrJAZ5*	*BrrJAZ21*	0.289814664	1.086552461	0.266728643
*BrrJAZ5*	*BrrJAZ24*	0.280436303	0.960001735	0.292120621
*BrrJAZ6*	*BrrJAZ13*	0.310897251	0.733513629	0.42384659
*BrrJAZ6*	*BrrJAZ18*	0.110131912	0.396713981	0.277610363
*BrrJAZ6*	*BrrJAZ14*	0.14080764	0.334597172	0.420827347
*BrrJAZ6*	*BrrJAZ20*	0.267411244	0.900685705	0.2968974
*BrrJAZ6*	*BrrJAZ23*	0.257072338	0.772968711	0.332577935
*BrrJAZ7*	*BrrJAZ26*	0.116400838	0.273732217	0.425236165
*BrrJAZ8*	*BrrJAZ9*	0.111605416	0.331472495	0.336695858
*BrrJAZ8*	*BrrJAZ10*	0.118162892	0.37303204	0.316763384
*BrrJAZ9*	*BrrJAZ10*	0.12305289	0.377358016	0.326090569
*BrrJAZ9*	*BrrJAZ19*	0.291372087	0.838022129	0.347690206
*BrrJAZ9*	*BrrJAZ22*	0.26883518	0.838762329	0.320514132
*BrrJAZ10*	*BrrJAZ19*	0.23344143	0.996471539	0.234268036
*BrrJAZ10*	*BrrJAZ22*	0.318746327	1.279382087	0.249140839
*BrrJAZ12*	*BrrJAZ17*	0.318031446	1.101430119	0.288744098
*BrrJAZ12*	*BrrJAZ21*	0.204272996	0.525827779	0.388478898
*BrrJAZ12*	*BrrJAZ24*	0.153074029	0.338528791	0.452174331
*BrrJAZ13*	*BrrJAZ20*	0.070342052	0.288061157	0.244191383
*BrrJAZ13*	*BrrJAZ23*	0.080141361	0.250173116	0.320343616
*BrrJAZ14*	*BrrJAZ18*	0.15528538	0.41123089	0.377611175
*BrrJAZ14*	*BrrJAZ20*	0.29053249	1.268013748	0.229124085
*BrrJAZ14*	*BrrJAZ23*	0.248347983	0.73705293	0.336947284
*BrrJAZ15*	*BrrJAZ16*	0.198282879	0.375581466	0.527935738
*BrrJAZ17*	*BrrJAZ21*	0.282206446	1.043439886	0.27045779
*BrrJAZ17*	*BrrJAZ24*	0.291820117	0.911871636	0.320023241
*BrrJAZ18*	*BrrJAZ20*	0.31821192	0.885669134	0.359289839
*BrrJAZ18*	*BrrJAZ23*	0.266567652	0.906244161	0.294145511
*BrrJAZ19*	*BrrJAZ22*	0.073378232	0.297500039	0.246649488
*BrrJAZ20*	*BrrJAZ23*	0.074057142	0.328109232	0.225708803
*BrrJAZ21*	*BrrJAZ24*	0.148592316	0.368754789	0.402956979
*BrrPPD1*	*BrrPPD2*	0.120953239	0.414612394	0.291726058
*BrrTIFY1*	*BrrTIFY3*	0.094257565	0.31859982	0.295849399
*BrrZML1*	*BrrZML3*	0.147663945	0.3741068	0.394710669
*BrrZML2*	*BrrZML4*	0.071414127	0.219683278	0.325077664
*BrrZML2*	*BrrZML5*	0.174472909	0.774718739	0.225208066

Ka: non-synonymous substitution rate; Ks: synonymous substitution rate; Ka/Ks: the average number of non-synonymous sites.

In the turnip genome, the ka/ks values of 44 duplicated *JAZ* gene pairs were lower than 1, suggesting that *JAZ* family genes evolved mainly under the influence of purifying selection.

The turnip *JAZ* family genes were distributed on 10 chromosomes, of which chromosome 2 (14), chromosome 7 (16), and chromosome 8 (15) had the highest number of homologous genes. *BrrJAZ6* of chromosome 2, *BrrJAZ20* of chromosome 8, and *BrrJAZ23* of chromosome 9 contained the highest number (5) of homologous genes in the turnip genome, while *BrrTIFY2* had no homologous genes in the turnip genome.

**Figure 6:**
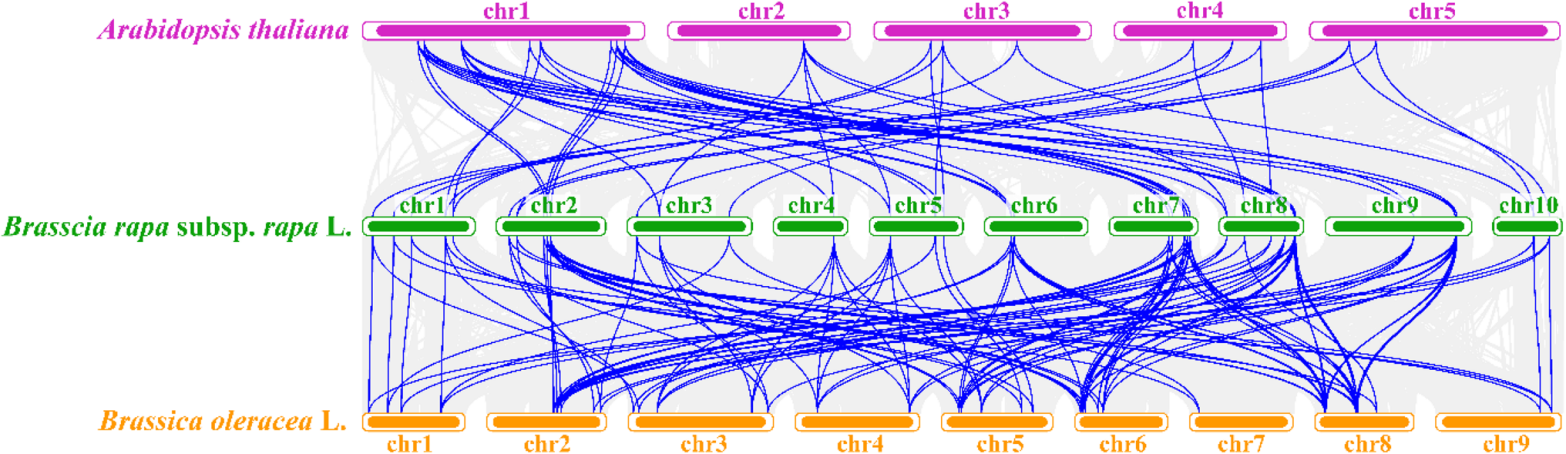
Collinear association of *Arabidopsis thaliana*, *Brassica Oleracea* and *Brassica rapa* subsp. *rapa* (turnip). Blue lines indicate duplicated *JAZ* gene pairs between *Arabidopsis*, *B. oleracea*, and turnip.

To infer the evolutionary relationship of *JAZ* genes among different species, the genomes of *A. thaliana*, *B. oleracea*, and turnip were analyzed by collinearity (Fig. 6). We detected many collinear blocks between their genomes. A total of 54 homologous *JAZ* gene pairs existed between the *A. thaliana* and turnip genomes. The homologous fragments between the two species were mainly distributed on chromosome 1 of *A. thaliana*, with 31 *JAZ* gene pairs. Chromosome 8 of turnip contained 10 homologous gene pairs.

A total of 121 pairs of *JAZ* genes between the *B. oleracea* and turnip genomes were covalently related. Homologous segments containing more pairs between species were mainly found on chromosomes 7 and 8 of turnip, containing 22 and 21 homologous pairs, respectively. Correspondingly, on chromosomes 6 and 8 of *B. oleracea*, containing 20 and 24 *JAZ* homologous pairs, respectively.

### Transcriptome analysis of *JAZ* family genes in turnip

**Figure 7:**
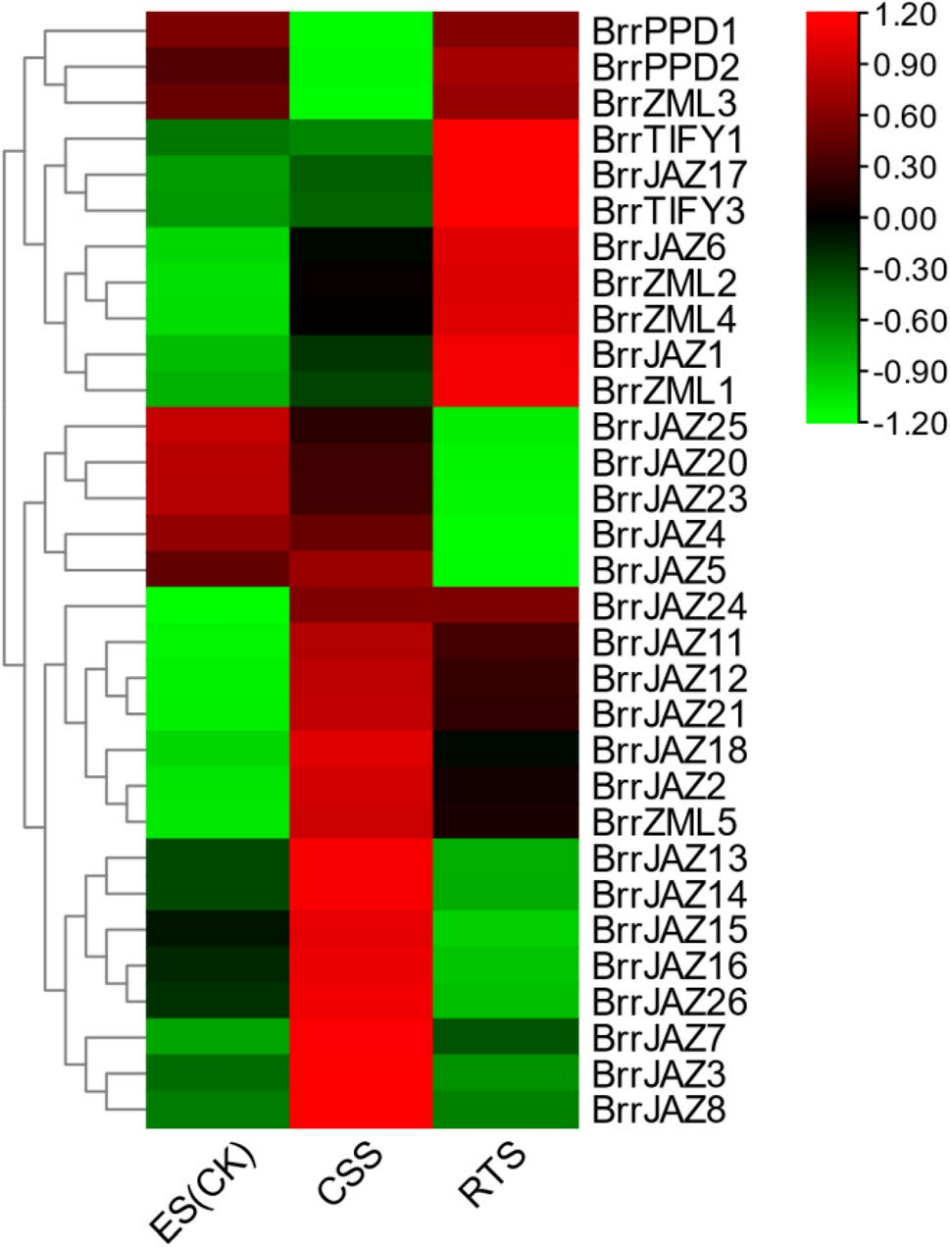
Expression of *BrrJAZ* gene family in different development stages of fleshy roots. ES, early stage before cortex splitting. CSS, cortex splitting stage. RTS, secondary root thickening stage. FPKM values of *BrrJAZ* genes were transformed by log2 and the heatmap was constructed by TBtools.

To explore the expression of *JAZ* family genes of turnip involved in tuberous root development, we analyzed the transcriptomic data published by Li et al. (Fig. 7). The expression patterns of turnip *JAZ* family genes in the three developmental periods could be distinguished. A total of five genes, including *BrrJAZ9*, *BrrJAZ10*, *BrrJAZ19*, *BrrJAZ22*, and *BrrTIFY2*, had no detectable expression. Most members of the *TIFY* and *ZML* subfamilies have close gene expression patterns, suggesting similar functions in the processes involved in tuberous root development. The diverse expression patterns of *JAZ* family genes in the three periods suggest that these members play more enriched functions in participating in the development of turnip tuberous roots.

### Expression analysis of *JAZ* family genes in turnip under abiotic stress and exogenous phytohormone treatment

**Figure 8:**
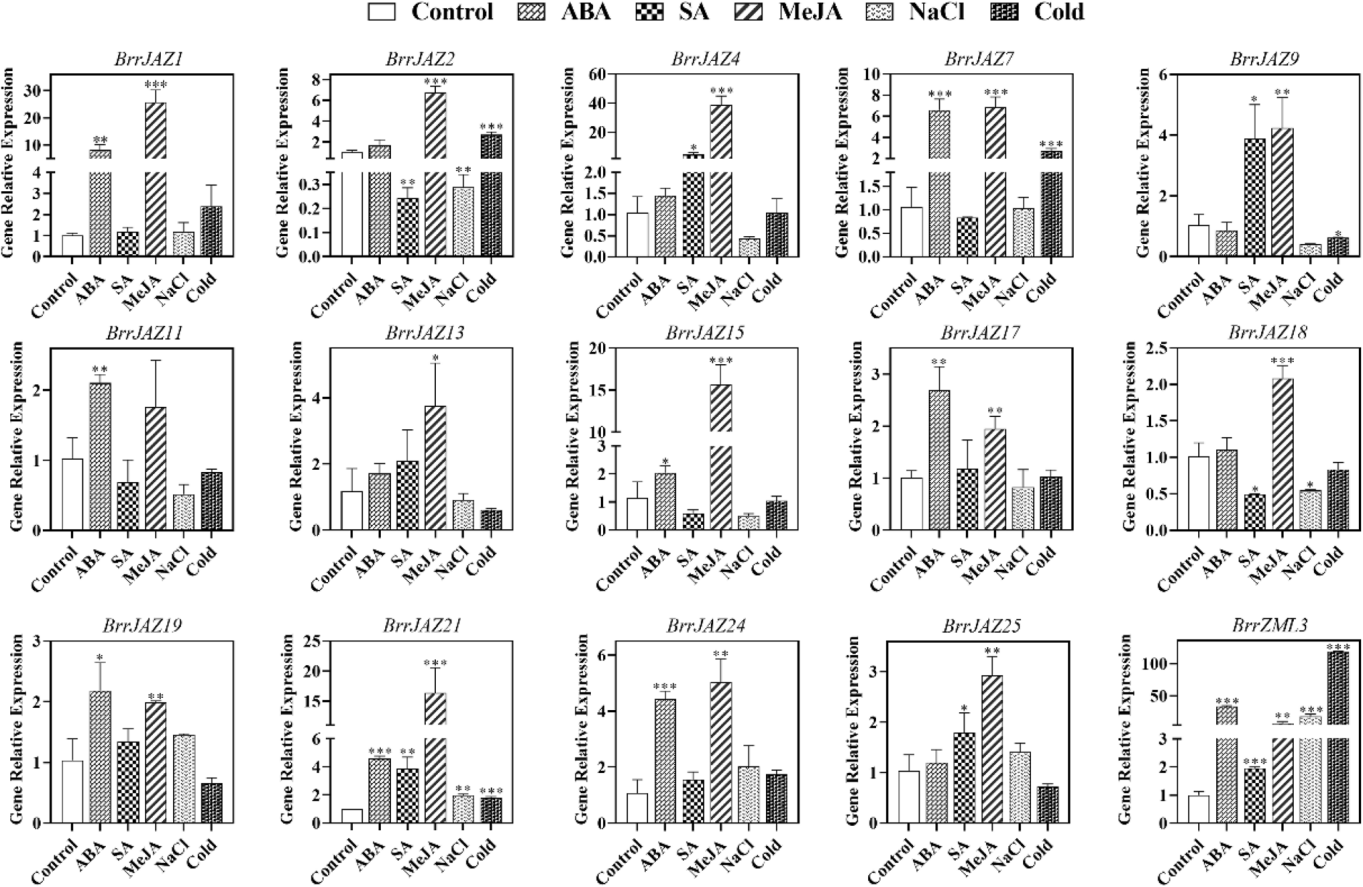
Expression of *BrrJAZ* family genes under abiotic stress and exogenous hormones. Error bars indicate standard deviation, and asterisks indicate significant differences between the control and treatments, *P < 0.05, **P < 0.01, ***P < 0.001.

To understand the expression pattern of *JAZ* family genes of turnip under different exogenous phytohormone and abiotic stress treatments, the leaves of turnip treated with ABA, SA, MeJA, salt stress, and low-temperature stress for 24 h were collected in this study, and the expression of *JAZ* family genes in each treatment was detected by qRT-PCR (Fig. 8). We found that *BrrJAZ21* and *BrrZML3* responded to all treatments. In all treatments, the expression of the above two genes was significantly different from the control.

The analysis of expression data showed that most of the *JAZ* family genes were up-regulated under exogenous ABA treatment. Among the 15 *JAZ* family genes tested, the expression of *BrrJAZ1*, *7*, *11*, *15*, *17*, *19*, *21*, *24*, and *BrrZML3* were significantly up-regulated.

After exogenous SA treatment, the 15 *JAZ* genes exhibited distinct expression patterns. The expression levels of *BrrJAZ4*, *9*, *21*, *25* and *BrrZML3* were significantly up-regulated compared with the control group, while the expression of *BrrJAZ2* and *BrrJAZ18* were significantly down-regulated.

Exogenous MeJA treatment increased the expression of the 15 *JAZ* family genes detected in the leaves of turnip seedlings. Except for the gene expression of *BrrJAZ11*, which was not significantly different from the control group, the gene expression of the other 14 genes was significantly increased compared with the control group.

After salt stress treatment, the gene expression of *BrrJAZ2* and *BrrJAZ18* was significantly lower than that of the control, while *BrrJAZ21* and *BrrZML3* were significantly higher than that of the control.

We also analyzed the expression of *JAZ* genes in turnip under low temperature stress. The expression of *BrrJAZ2*, *7*, 21 and *BrrZML3* were significantly up-regulated after low temperature stress treatment, whereas *BrrJAZ9* was significantly lower than the control group.

## Discussion

*JAZ* is a plant-specific gene family with prominent roles in the regulation of many physiologic processes in plant growth and stress response through JA signalings, such as seed germination^36^, flower development^37^, response to salt, drought, high temperature, wound, and diseases^18,38,39^. However, few studies have been reported on the functional analysis of turnip *JAZ* gene family members. Therefore, in this study, we identified the *JAZ* family genes in the turnip genome and analyzed the sequence information of each member to investigate their expression patterns under abiotic stresses and exogenous phytohormone treatments.

*JAZ* family genes were widely identified in some Brassica crops. Previous research identified 36, 38, 36, and 36 *JAZ* genes in *B. rapa* L.^40^, *B. juncea* var. *tumida*^41^, *B. napus* L.^42^, and *B. oleracea* var. *capitata*^30^, respectively. In the present study, we identified 36 members of *JAZ* genes in the turnip genome. This result suggested that the number of *JAZ* family genes is conservative and has not changed significantly during the process of species formation. The composition of the turnip *JAZ* gene family members was more similar to that of other dicotyledons. The JAZ protein sequences of the tea plant^43^, *Arabidopsis*^44^, tomato^45^, and Brassica^30,40–42^ all contain members of the TIFY, JAZ, and PPD subfamilies, and members of these three subfamilies have been identified in the JAZ family protein sequence of turnip. *JAZ* family genes have numerous members and are likely to perform different functions in response to adversity stress.

Gene duplications contribute to the expansion of new gene family members and provide an opportunity for novel functions in the evolution of the plant genome. Therefore, investigating gene duplication can help us understand the evolution of genes and species. Whole-genome duplication, segmental duplication, and tandem duplication are the three main pathways of gene duplication^44^. Previous studies showed that no tandem duplication events of *JAZ* family genes were found in the *B. rapa* L. and *B. juncea* var. *tumida*^40,41^, whereas two pairs of tandem duplication genes were identified in *Brassica oleracea* var. *capitata*^30^. In concert with the findings in *B. oleracea* var. *capitata*, we also detected only two pairs of tandem duplication *JAZ* genes in the turnip genome. Our results indicate that whole-genome duplication or segmental duplication were predominant duplication events for *JAZ* genes.

To determine the selective evolutionary pressure for *BrrJAZ* genes differentiation after duplication, Ka and Ks values for duplicated *BrrJAZ* gene pairs were calculated using the Ka/Ks calculator. In general, Ka/Ks = 1 indicates neutral selection, Ka/Ks > 1 indicates positive selection, and Ka/Ks < 1 indicates purification selection^46^. Our results showed that the Ka/Ks value of each duplicated *BrrJAZ* gene pair was less than 1, which indicated the purification selection during evolution. Similarly, the Ka/Ks values of duplicated homologous gene pairs in the *JAZ* gene family of *Solanum lycopersicum*^45^, *Phyllostachys edulis*^47^, *B*. *oleracea* var. *capitata*^30^, and *Petunia*^48^ were less than 1, indicating that the *JAZ* genes of these species were subjected to strong purifying selection, which may have led to functional conservation or pseudogenization. In contrast, in the maize genome, three repetitive blocks had Ka/Ks > 1, indicating accelerated evolution under positive selection^45^.

JAZ proteins may be involved in the root development process in plants. Han et al. found that JAZ proteins interact with RHD6/RSL1, a transcription factor that regulates root growth, repressing the transcriptional function of RHD6 and interfering with the interaction between RHD6 and RSL1, suggesting that JAZ proteins play an important role in *Arabidopsis* root development^49^. In this study, after mining transcriptome data of Li et al. during the development of turnip tuberous roots, we found that *JAZ* family genes varied greatly during three periods of turnip tuberous root growth, indicating that *JAZ* genes are likely to be involved in the development of turnip tuberous roots, and this will be used as an entry point for validation in future studies^50^.

Plants regulate responses to growth, development and environmental stresses at the transcriptional level. Therefore, we analyzed the expression of *JAZ* family genes in turnips under different stress conditions. Our results showed that most *BrrJAZs* responded significantly to abiotic stress and/or exogenous phytohormone treatments, which is in agreement with the results obtained in other Brassica crops^30,40–42^.

Many studies have demonstrated that exogenous JAs treatment can strongly induce the expression of *JAZ* genes. Saha et al. found that the expression of *JAZ* genes was significantly up-regulated by exogenous JA treatment, increased 15-fold to 800-fold compared with the control^40^. Liu et al. found that all *BoJAZ* family genes were up-regulated after exogenous MeJA treatment, and the expression of 8 genes showed a highly significant increased, which was more than fivefold higher than the control^30^. Our results were in agreement with the findings above. We found that MeJA treatment increased the expression of *JAZ* family genes. The expression of *BrrJAZ4* was elevated the most compared to the control group, reaching 36.4-fold.

Different expression patterns of *JAZ* family genes emerged after exogenous SA treatment. A total of seven genes showed significant differences in expression from the control. Among them, two genes were significantly down-regulated in expression, while five genes were significantly up-regulated in expression. Liu et al. found that the expression of *JAZ* family genes showed insignificant changes after induction by exogenous SA, and only 3 of the 22 *JAZ* genes were up-regulated^30^. This suggests that although closely related species have similar numbers of *JAZ* family genes and relatively close phylogenetic relationships, they may have different functions.

*JAZ* genes are transcriptional repressors of jasmonate-responsive genes, which contain two highly conserved sequence regions: N-terminal ZIM/TIFY structural domain mediates homomeric and heteromeric interactions between most JAZ proteins. C-terminal JAS domain plays a key role in destabilizing JA-Ile response repressors^51^. Abiotic stresses such as low-temperature, drought, and salt stress can induce up-regulation of *JAZ* gene expression in rice. Moreover, overexpression of *OsTIFY11a* significantly increased tolerance to salt and dehydration stresses^26^. In grapes, 11 *TIFY* genes were found to be responsive to osmotic stress and low-temperature stress^28^. Our findings were slightly different from the above studies. We found that most of the turnip *JAZ* genes were not significantly changed under salt stress treatment. Among the 15 genes tested, only two genes were significantly up-regulated and two genes were significantly down-regulated. Moreover, the qPCR data revealed that only a small number of genes were up-regulated in expression under low-temperature stress, while most *JAZ* family genes did not show significant differences in expression compared to the control. Taken together, the above qPCR data analysis showed that the *BrrZML3* gene responded positively to all exogenous plant hormone treatments and abiotic stress treatments. This is most likely related to its gene structure.

## Conclusions

In this study, we identified 36 *JAZ* genes from the turnip genome and classified them into four subfamilies. They were unevenly distributed among 10 chromosomes. Gene structure and conserved motifs of *BrrJAZs* were similar within the subfamilies, but the differences between the subfamilies were large. Although the proteins varied in length, MW, and pI, all contained a conserved TIFY or JAS domain. Phylogenetic and collinearity analysis provided some valuable clues to the evolutionary characteristics of *BrrJAZ* genes. Expression analysis suggested *JAZ* genes may be involved in the formation of turnip tuberous root, and they also participated in the response to salt and low-temperature stress. Several *BrrJAZ* genes were also responsive to ABA, SA and MeJA treatment. Overall, our findings will help understand the biological functions of the *BrrJAZ* genes in turnip.

## Supplementary Information

Supplementary Table S1.

Supplementary Table S2.
